# Prevalence and Indicators of Tooth Wear among Chinese Adults

**DOI:** 10.1371/journal.pone.0162181

**Published:** 2016-09-01

**Authors:** Zhao Wei, Yangge Du, Jing Zhang, Baojun Tai, Minquan Du, Han Jiang

**Affiliations:** The State Key Laboratory Breeding Base of Basic Science of Stomatology (Hubei-MOST) and Key Laboratory of Oral Biomedicine Ministry of Education, School & Hospital of Stomatology, Wuhan University, Wuhan City, China; The Ohio State University, UNITED STATES

## Abstract

Numerous epidemiological studies have focused on the prevalence and related indicators of tooth wear. However, no sufficient studies have been conducted with Chinese adults. The purpose of this study was to assess the prevalence of tooth wear and identify related indicators among adults aged 36 to 74 years in Wuhan City, P.R. China. A cross-sectional and analytic study was conducted with 720 participants, aged 35–49 yrs and 50–74 yrs, in 2014. Each age group included 360 participants, of which 50% were males and 50% were females. All participants completed a questionnaire before examination. Tooth wear was assessed using the modified Basic Erosive Wear Examination (BEWE) index. The data were analyzed using the chi-square test and binary logistic regression analysis. The prevalence of tooth wear was 67.5% and 100% in the 35–49 and 50–74 age groups, respectively. The prevalence of dentin exposure was 64.7% and 98.3%, respectively. A significantly higher prevalence of tooth wear and dentin exposure was found in the 50–74 yr group than in the 35–49 yr group (p < 0.05). Critical indicators of tooth wear and dentin exposure included high frequency of acidic drinks and foods consumption, low socio-economic status, and unilateral chewing. The frequency of changing toothbrushes and the habit of drinking water during meals were associated with tooth wear. In addition, the usage of hard-bristle toothbrushes and consuming vitamin C and aspirin were found to be linked with dentin exposure. In conclusion, the prevalence of tooth wear and dentin exposure observed in Chinese adults was high, and the results revealed an association between tooth wear and socio-behavioral risk indicators.

## Introduction

Tooth wear is generally defined as the irreversible chronic loss of dental hard tissues caused by mechanical and/or chemical processes without the involvement of bacterial plaque[1, 2]. To some degree, tooth wear is a physiological and age-dependent process. However, a pathological status may be reached when the teeth are so worn that their appearance is affected or their functionality is impaired[3]. The clinical appearance of tooth wear always involves a complex interaction of biological, tribological, mechanical, and chemical factors. Based on the etiological factors, tooth wear has traditionally been divided into the following three types: attrition (wear produced physiologically through tooth-tooth contact), abrasion (wear produced through interaction between the teeth and foreign objects) and erosion (dissolution of the dentin hard tissue by acidic substances)[4].

In recent years, many epidemiological studies have focused on the prevalence and the etiology of tooth wear in adults. The prevalence of tooth wear is high and varies widely in different parts of the world[5]. In Germany, among 836 people, the prevalence of tooth wear with dentin exposure was 23.4%[6]. In Japan, among 1108 participants aged 15–89 yrs, 26.1% had signs of erosive wear[7]. A recent study from Israel showed a prevalence of 61.9% in adults aged 55–60 yrs[8]. Furthermore, in Northern India, 71.1% of 965 male fertilizer factory workers aged 19–58 yrs had tooth wear[9].

In China, the data are not very well established. Zhang *et al*. reported the prevalence of tooth wear among 12- and 15-year-old adolescents from Central China[10]. Based on the tooth site, Liu et al. reported that the prevalence of tooth wear in aging people in Northwest China ranged from 85.51% to 100.0%[11]. However, data regarding the prevalence of tooth wear in Chinese adults are scarce. The purpose of the present study was to assess the prevalence of tooth wear and identify related indicators among Chinese adults aged 35–49 and 50–74 yrs.

## Materials and Methods

The study protocol was approved by the Ethics Committee of the School & Hospital of Stomatology of Wuhan University, Wuhan City, P.R. China.

### Sampling Procedure

**Fig 1:**
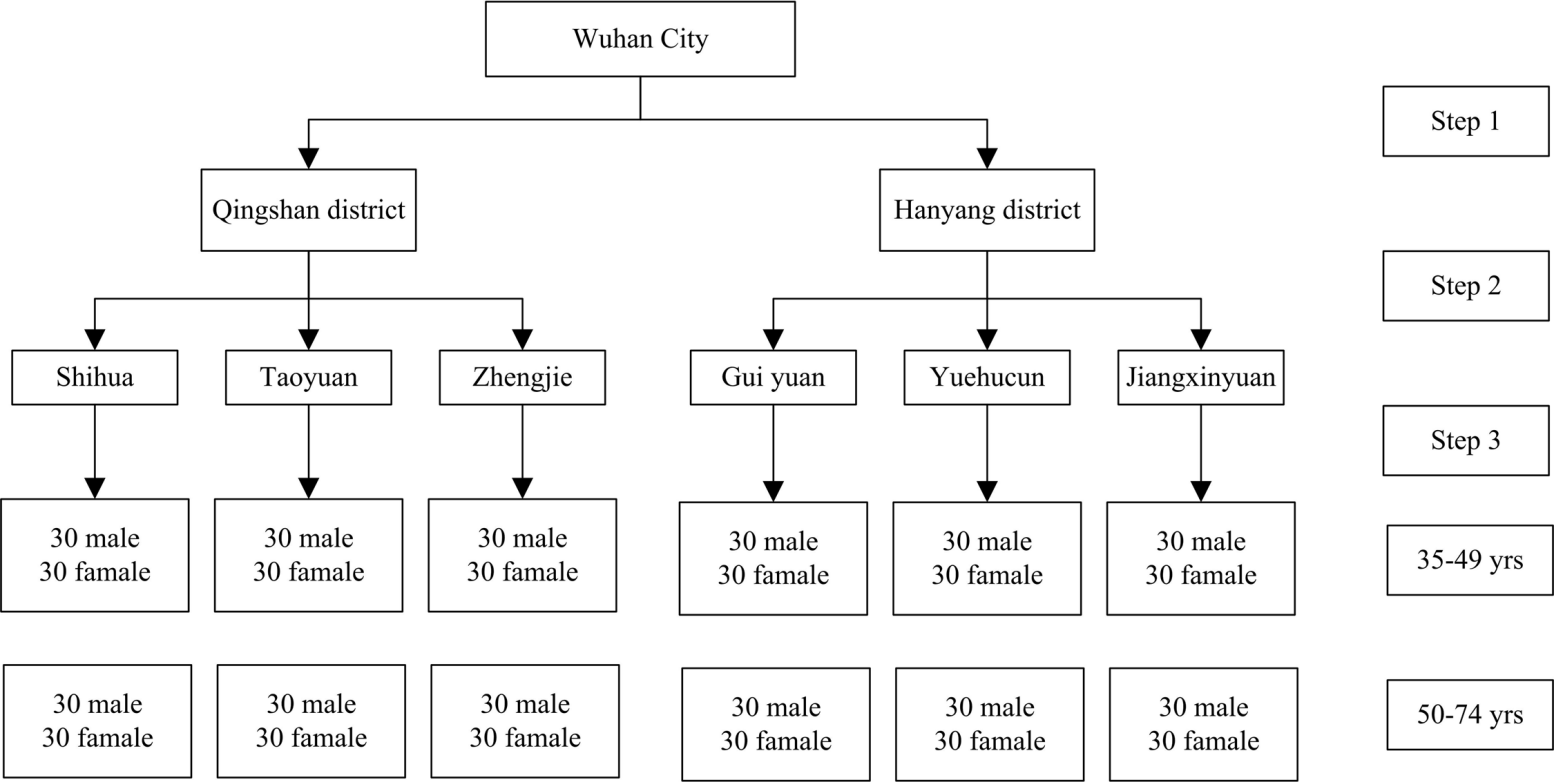
Schematic illustration of multistage sampling.

The duration of this cross-sectional study was from October 2014 to December 2014. The survey employed a multistage stratified sampling method to obtain a representative sample of adults aged 35–49 yrs and 50–74 yrs in Wuhan City. A minimum sample size of 322 adults was required for each age group, assuming a tooth wear prevalence of 30% [7] with a 95% confidence interval (CI). This sample size was also required to contain a 5% acceptable margin of error and an alpha level of 0.05. Ultimately, each group included 360 participants. The participants were randomly chosen from 6 communities in Wuhan. Each age group was divided into male and female subgroups. In brief, during the first stage, two districts (the Qingshan District and the Hanyang District) were chosen randomly from the 13 districts in Wuhan City. Then, three communities were randomly chosen within each selected district. During the third stage, 60 participants from each of two age groups (50% male and 50% female) were selected randomly from each resident community (Fig 1). To obtain relatively more accurate data, the adults who were included in this study had to have lived in the local communities for more than 6 months. At the beginning of this survey, each participant received, read and signed an informed consent form that explained the purpose and procedures of the study. Individuals who withheld consent were not included in the study. Participants were also excluded if they had serious diseases or were receiving drug treatments for any oral diseases.

### Interview

Before the clinical examinations were performed, the participants underwent interviews via a questionnaire. The questionnaire included items related to the following topics: the frequency of acidic foods and drinks consumption (fresh fruit, fruit juice, vegetable juice, pickled vegetables, carbonated drinks, coffee, wine, vinegar), medicine usage (vitamin C, aspirin), chewing habits, systemic diseases, tooth-brushing habits, and family socio-economic status.

### Clinical Examination

Two dentists performed the clinical examinations. The examiners were asked to undergo an initial calibration trial on a group of participants under the guidance of Bartlett[12] prior to the formal examinations. The inter-examiner reliability was evaluated using the kappa test; after calibration, the kappa score was above 0.70. During the survey period, approximately 5% of the participants (n = 6) were randomly chosen each day to be re-examined to confirm the intra-examiner reliability. The intra-examiner kappa values of the two examiners were 0.80 and 0.82.

All of the clinical examinations were performed in portable dental chairs under artificial light. Two experienced nurses were responsible for recording the data. The teeth were dried with cotton wool rolls. Food residue and soft matter were removed before assessment. The modified Basic Erosive Wear Examination (BEWE) index (Table 1) was used to record tooth wear and dentin exposure. Using this scoring system, the buccal, cervical, lingual/palatal and occlusal surfaces of all permanent teeth present, except the third molar, were examined. In this procedure, each surface was given the following two scores: one for wear on the enamel and the other for dentin exposure.

**Table 1:** Modified Basic Erosive Wear Examination (BEWE) index.

Score	Tooth wear	Dentin exposure
0	No tooth wear	Limited to enamel
1	Initial loss of surface texture	Spread to dentin
2	Distinct defect, hard tissue loss<50% of the surface area	
3	Hard tissue loss ≥ 50% of the surface area	
8	Orthodontic appliances, caries or restoration ≥ 25% of the surface area, partial eruption, trauma, crown, unable to be accessed	
9	Missing	

### Statistical Analysis

Data analysis was accomplished using SPSS version 21 (IBM; Chicago, IL, USA) for Windows. The maximum BEWE score and the maximum dentin exposure (DE) score recorded for all tooth surfaces (except 8 and 9) were calculated for each participant. For further descriptive statistical analysis of the distribution of tooth wear and dentin exposure, percentages were calculated and subdivided for each age group and gender subgroup. The prevalence of tooth wear was the proportion of participants with BEWE = 3[13], and the prevalence of dentin exposure was the proportion of participants with DE = 1. The relationship between tooth wear/dentin exposure and the questionnaire items was evaluated using the chi-square test and binary logistic regression analysis. Tooth wear and dentin exposure were analyzed separately. The dependent variables were defined as BEWE = 3 or DE = 1. All independent variables that were significant in the chi-square analysis were entered as candidates and included in the binary logistic regression analysis. Odds ratios (ORs) with 95% confidence intervals (95% CI) were calculated in the logistic regression model to evaluate the connection between the dependent variables and the potential risk indicators.The statistical significance was set at 0.05.

## Results

A sample of 720 participants (360 males and 360 females) was equally distributed among the two age groups (n = 360). The distribution of BEWE scores in the 35–49 yr stratum and the 50–74 yr stratum based on gender differences is shown in Table 2; the prevalence of tooth wear for the two groups was 67.5% and 100%, respectively. The prevalence of dentin exposure changed from 64.7% for the middle-aged adults to 98.3% for the older group. The prevalence of tooth wear and dentin exposure increased with age(p<0.001). No significant difference was observed with regard to gender.

**Table 2:** The prevalence of tooth wear and dentin exposure according to gender and age in Wuhan, China.

	N	Tooth wear	Dentin exposure
		N(%)	N(%)
35–49 yrs			
Male	180	117(65.2)	123(68.5)
Female	180	126(69.8)	110(60.9)
Total	360	243(67.5)	233(64.7)
50–74 yrs			
Male	180	180(100)	176(97.8)
Female	180	180(100)	178(98.9)
Total	360	360(100)	354(98.3)

In Table 3 and Table 4, the data from the 720 participants enrolled in this investigation was assessed using the chi-square test, which revealed associations between the percentages of tooth wear (Table 3) and dentin exposure (Table 4) and various factors. Tooth wear and dentin exposure were associated with age, frequency of acidic drinks and foods consumption, habit of holding drinks in mouth, drinking water during meals, taking vitamin C, taking aspirin, unilateral chewing, frequency of changing toothbrushes, frequency of tooth brushing, socio-economic status and hardness of toothbrush bristles (p<0.05). The duration of tooth brushing, gastroesophageal reflux disease, bruxism and gastricism were associated only with dentin exposure (p<0.05). No significant difference was found for gender, drinking before sleep, frequency of tea consumption, frequency of swimming in summer, clenching teeth automatically and horizontal brushing.

**Table 3:** The relationship between tooth wear and associated factors among the study population.

	N	Tooth wear		P-value ^a^
		BEWE = 3	%	
Age				<0.001
35–49 yrs	360	243	67.5	
50–74 yrs	360	360	100	
Gender				0.381
Male	360	298	82.5	
Female	360	305	85.0	
Frequency of acidic drinks and foods consumption				<0.001
Low (scores 9–20)	230	168	73.0	
Medium (scores 21–28)	256	222	86.7	
High (scores 29–45)	234	213	91.0	
Drinking before sleep				0.145
Never/Rarely	639	538	84.2	
Sometimes	64	49	76.6	
Often	17	16	94.1	
Holding drinks in mouth				0.008
Never	615	525	85.4	
Rarely	82	58	70.7	
Sometimes	21	18	85.7	
Often	2	2	100	
Drinking water during meals				<0.001
Never	258	238	92.2	
Rarely	187	151	80.7	
Sometimes	170	130	76.5	
Often	105	84	80.0	
Frequency of tea consumption				0.404
>once daily	198	160	80.8	
1–6 times weekly	85	73	85.9	
≤3 times monthly	437	370	84.7	
Frequency of swimming in summer				0.645
Never/Rarely	707	593	83.9	
Sometimes	12	9	75.0	
Often	1	1	100	
Taking vitamin C				0.001
Never	514	447	87.0	
Rarely	133	102	76.7	
Sometimes	48	33	68.8	
Often	25	21	84.0	
Taking aspirin				0.013
Never	605	498	82.3	
Rarely	50	42	84.0	
Sometimes	15	13	86.7	
Often	50	50	100	
Gastroesophageal reflux disease				0.297
No	681	568	83.4	
Yes	39	35	89.7	
Gastricism				0.056
No	691	575	83.0	
Yes	29	28	96.9	
Clenching teeth automatically				0.831
Never/Rarely	660	551	83.5	
Sometimes	53	46	86.8	
Often	7	6	85.7	
Bruxism				
Never/Rarely	670	562	83.9	0.941
Sometimes	39	32	82.1	
Often	11	9	81.8	
Unilateral chewing				0.003
No	383	306	79.9	
Yes	337	297	88.1	
Frequency of tooth brushing				0.005
≥2 times daily	522	423	81.0	
Once daily	195	177	90.8	
<once daily	3	3	100	
Frequency of changing tooth brushes				0.003
1 month	118	91	77.1	
2 months	192	152	79.2	
3 months or more	410	360	87.8	
Social-economic class				<0.001
Low (scores 1–3)	164	153	93.3	
Medium (scores 4–6)	310	258	83.2	
High (scores 7–10)	246	192	78.0	
Duration of tooth brushing				0.148
≥1 min	651	541	83.1	
<1 min	69	62	89.9	
Tooth brush bristle				0.005
Soft bristle	394	316	80.2	
Medium bristle	218	187	85.8	
Hard bristle	108	100	92.6	
Horizontal brushing				0.052
No	138	108	85.1	
Yes	582	495	78.3	

BEWE, Basic Erosive Wear Examination

^a^ P values were calculated using the χ^2^ test.

**Table 4:** The relationship between dentin exposure and associated factors among the study population.

	N	Dentin exposure		P-value ^a^
		DE = 1	%	
Age				<0.001
35–49 yrs	360	233	64.7	
50–74 yrs	360	354	98.5	
Gender				0.437
Male	360	212	58.7	
Female	360	221	61.6	
Frequency of acidic drinks and foods consumption				<0.001
Low (scores 9–20)	230	86	37.4	
Medium (scores 21–28)	256	158	61.7	
High (scores 29–45)	234	189	80.8	
Drinking before sleep				0.063
Never/Rarely	639	402	69.9	
Sometimes	64	31	48.0	
Often	17	10	58.8	
Holding drinks in mouth				<0.001
Never	615	404	65.7	
Rarely	82	23	28.0	
Sometimes	21	6	28.6	
Often	2	0	0.0	
Drinking water during meals				<0.001
Never	258	210	81.4	
Rarely	187	95	50.8	
Sometimes	170	70	41.2	
Often	105	58	55.2	
Frequency of tea consumption				0.108
>once daily	198	107	54.0	
0030	85	55	64.7	
≤3 times monthly	437	271	62.0	
Frequency of swimming in summer				0.075
Never/Rarely	707	429	60.7	
Sometimes	12	4	33.3	
Often	1	0	0.0	
Taking vitamin C				<0.001
Never	514	345	67.1	
Rarely	133	54	40.6	
Sometimes	48	17	35.4	
Often	25	17	68.0	
Taking aspirin				<0.001
Never	605	347	57.4	
Rarely	50	25	50.0	
Sometimes	15	12	80.0	
Often	50	49	98.0	
Gastroesophageal reflux disease				0.004
No	681	401	58.9	
Yes	39	32	82.1	
Gastricism				<0.001
No	691	406	58.8	
Yes	29	27	93.1	
Clenching teeth automatically				0.330
Never/Rarely	660	402	60.9	
Sometimes	53	28	52.8	
Often	7	3	42.9	
Bruxism				0.016
Never/Rarely	670	412	61.5	
Sometimes	39	15	38.5	
Often	11	6	54.5	
Unilateral chewing				<0.001
No	383	201	52.5	
Yes	337	232	68.8	
Frequency of tooth brushing				<0.001
≥2 times daily	522	273	52.3	
Once daily	195	158	81.0	
<once daily	3	2	66.7	
Frequency of changing tooth brushes				<0.001
1 month	118	70	59.3	
2 months	192	94	49.0	
3 months or more	410	269	65.6	
Socio-economic class				<0.001
Low (scores 1–3)	164	137	83.5	
Medium (scores 4–6)	310	205	66.1	
High (scores 7–10)	246	91	37.0	
Duration of tooth brushing				0.028
≥1 min	651	383	58.8	
<1 min	69	50	72.5	
Tooth brush bristle				<0.001
Soft bristle	394	199	50.5	
Medium bristle	218	142	65.1	
Hard bristle	108	92	85.2	
Horizontal brushing				0.563
No	138	80	60.7	
Yes	582	353	58.0	

DE, dentin exposure

^a^ P values were calculated using the χ^2^ test.

Table 5 shows the results of the binary logistic regression analysis for tooth wear (BEWE = 3), and Table 6 shows the results for dentin exposure (DE = 1). The indicated factors for tooth wear were high frequency of acidic drinks and foods consumption (OR = 2.10, p<0.001), low socio-economic status (OR = 3.91, p = 0.049), frequency of changing toothbrushes (OR = 2.08, p = 0.022), unilateral chewing (OR = 1.67, p = 0.021) and drinking water during meals (OR = 2.48, p = 0.011). Regarding dentin exposure, the results of the logistic regression analysis demonstrated a higher prevalence among the participants from a low socio-economic class (OR = 3.88, p<0.001) and for those with a high frequency of acidic drinks and foods consumption (OR = 3.90, p<0.001). In addition, participants who chewed unilaterally, took vitamin C or aspirin, or used a hard-bristled toothbrush also tended to have a high probability of experiencing dentin exposure.

**Table 5:** Binary logistic regression analyses of odds for tooth wear among Chinese adults.

	P-value ^a^	Adjusted OR	95% CI	
			Lower	Upper
Frequency of acidic drinks and foods consumption	0.001			
Low (scores 9–20)				
Medium (scores 21–28)	0.001	2.58	1.462	4.535
High (scores 29–45)	0.003	2.10	1.291	3.408
Socio-economic class	0.049			
High (scores 7–10)				
Medium (scores 4–6)	0.014	1.40	0.913	2.133
Low (scores 1–3)	0.039	3.91	1.977	7.739
Frequency of changing tooth brushes	0.022			
1 month				
2 months	0.397	1.29	0.715	2.332
3 months or more	0.011	2.08	1.184	3.636
Unilateral chewing	0.021			
No				
Yes	0.021	1.67	1.082	2.578
Drinking water during meals	0.011			
Never				
Rarely	0.009	0.44	0.235	0.810
Sometimes	0.001	0.37	0.197	0.680
Often	0.012	0.40	0.198	0.818

Adjusted OR, adjusted odds ratio; CI, confidence interval

^a^ P-values were calculated using binary logistic regression analyses.

**Table 6:** Binary logistic regression analyses of odds for dentin exposure among Chinese adults.

	P-value ^a^	Adjusted OR	95% CI	
			Lower	Upper
Frequency of acidic drinks and foods consumption	<0.001			
Low (scores 9–20)				
Medium (scores 21–28)	<0.001	2.10	1.354	3.262
High (scores 29–45)	0.01	3.90	2.368	6.428
Taking vitamin C	0.017			
Never				
Rarely	0.132	0.58	0.363	0.957
Sometimes	0.113	0.54	0.254	1.156
Often	0.013	2.28	0.821	6.343
Taking aspirin	0.008			
Never				
Rarely	0.766	0.89	0.433	1.853
Sometimes	0.099	3.60	0.787	16.454
Often	0.003	22.96	2.994	176.028
Unilateral chewing	0.005			
No				
Yes		1.71	1.174	2.480
Tooth brush bristle	0.009			
Soft bristle				
Medium bristle	0.064	2.68	1.395	5.142
Hard bristle	0.003	1.39	0.912	2.129
Socio-economic class	<0.001			
High (scores 7–10)				
Medium (scores 4–6)	<0.001	2.02	1.341	3.035
Low (scores 1–3)	0.001	3.88	2.211	6.807

Adjusted OR, adjusted odds ratio; CI, confidence interval

^a^ P-values were calculated using binary logistic regression analyses.

## Discussion

With increases in longevity and decreases in the rates of tooth loss, tooth wear has been perceived as a problem, especially among adults. This investigation is thought to be the first attempt to present the current tooth wear and dentin exposure situations among adults aged 35–49 and 50–74 yrs in China.

The accurate documentation of tooth wear severity is very important for epidemiological studies. Numerous indices have been presented to record tooth wear such as the Tooth Wear Index (TWI)[11, 14]. However, there is no universally accepted method for evaluation. The barriers to TWI in practice are that it is time consuming, and it is difficult to handle and reconcile both the clinical and experimental imperatives[15]. The Basic Erosive Wear Examination (BEWE) index provides a simple method to screen tooth wear and was designed by Bartlett *et al*. based on the basic periodontal examination (BPE) [12]. Previous studies have shown that this score had sufficient sensitivity and specificity and has a similar distribution to Tooth Wear Index scores[13, 16].

The prevalence of tooth wear was 67.5% in the 35–49 age group. It is important to note that this prevalence rate of tooth wear was higher than that reported in Israel[8], in London[17] and in Ireland[18] but lower than that reported in India[9] and in Northwest China[11]. The prevalence of tooth wear was 100% in the 50–74 age group, which is consistent with the data reported by Liu in a study in Northwest China[11]. The prevalence of dentin exposure was 64.7% in the 35–49 age group. Previous studies have reported that the prevalence of dentin hypersensitivity among adults ranged between 4% and 67.7%[19], which is consistent with our results. The prevalence of dentin exposure was 98.3% among the 50–74 age group in our study, which is higher than reported in a previous study[17,19]. This disparity may be related to differences in regions, age groups, evaluation criteria or living habits.

There is substantial evidence demonstrating that the consumption of acidic foods and drinks is a risk factor for tooth wear [20,21]. Additionally, a study in vitro showed that beverages had the potential to erode dentin hard tissue[22]. In this study, the high frequency of acidic drinks and foods consumption also exhibited high odds ratios, indicating a strong association with tooth wear and dentin exposure; as such, the results support the clinical conjecture and laboratory findings that indicate acidic foods and drinks cause erosion.

Socio-economic status may also contribute to tooth wear and dentin exposure. In the present study, a significantly higher tooth wear and dentin exposure prevalence was found in the lowest socio-economic category (OR = 3.91 and 3.88 for the 35–49 yr and 50–74 yr groups, respectively). This result is in accordance with several studies[10, 19, 23]. This connection between tooth wear and dentin exposure and socio-economic factors may be related to diet, living habits, awareness of oral health or the selection of prosthodontic methods. Further studies are needed to examine these connections.

Another factor associated with tooth wear and dentin exposure in adults was unilateral chewing (OR = 1.67 and 1.71 for the 35–49 yr and 50–74 yr groups, respectively). A study conducted by Hayato among patients with unilateral missing posterior teeth showed that there was no significant difference between unilateral chewers and bilateral chewers with normal dentition in mastication predominance[24]. Chewing side preference resulted in a higher bite force and greater occlusal contact, causing more tooth wear and dentin exposure on the preferred chewing side[25]. In this study, 46.8% of participants had a preferred chewing side; compared with bilateral chewers, they exhibited a 1.67-fold higher OR for tooth wear and a 1.71-fold higher OR for dentin exposure. Similar results were reported in an epidemiological study in which tooth wear was investigated in 12- and 15-year-old adolescents in Central China[10].

Taking vitamin C and aspirin were also found to be associated with dentin exposure in adults. These results are corroborated by other studies that observed an association between vitamin C and aspirin use and dental damage[10, 26–28]. Vitamin C has been proven to be more erosive than phosphoric acid and citric acid[29]. According to the literature, megadoses of vitamin C can cause a sustained drop in salivary pH to below 5.5, which is the critical point of enamel dissolution, for up to 25 minutes[30]. Aspirin is a type of soluble analgesic that can contribute to tooth wear because it contains acetylsalicylic acid. Grace *et al*. also revealed the connection between erosion and aspirin, which provided important support for our result[28]. Vitamin C and aspirin tablets were significantly associated with the development of erosion; therefore, doctors should guide patients to help them develop reasonable health behaviors in this regard.

This study also observed that the use of hard-bristled toothbrushes was related to dentin exposure. The type of pathological change caused by tooth brushing is abrasion. This finding is consistent with a previous laboratory study that confirmed the connection between the hardness of toothbrush bristles and tooth wear[31,32]. In addition, studies have indicated that a frequency of tooth brush changes of more than or equal to 3 months could increase the risk of tooth wear. This may be due to the changes of the hardness of the bristles.

Furthermore, the habit of drinking water during meals was found to be negatively associated with the occurrence of tooth wear in adults from China. This habit means that participants are used to drinking water at mealtimes instead of any other types of drinks such as soda, fruit juice, liquor, wine, beer, tea, coffee, or soups. This habit can help control the consumption of sugary or acidic drinks from the perspective of nutrition [33]. On the other hand, drinking water during meals helps with swallowing foods, reducing tooth attrition.

Factors such as gender, frequency of tooth brushing, duration of tooth brushing consumption of tea, systemic diseases, frequency of swimming in summer, clenching teeth automatically, horizontal brushing and drinking before sleep were also recorded in the questionnaire, but the results obtained in this study did not show a significant relationship for any of them.

It was evident from the study that tooth wear and dentin exposure occur with a high frequency among adults aged 35–74 yrs in China. A better understanding of the risk indicators associated with tooth wear and dentin exposure may lead to more effective interventions.

## Conclusions

The results of this cross-sectional study showed a high prevalence of tooth wear and dentin exposure among middle-aged and older adults in China. The study also analyzed the etiology of tooth wear and dentin exposure. A high frequency of acidic drinks and foods consumption, low socio-economic status, and unilateral chewing appeared to increase the risk of tooth wear and dentin exposure. The frequency of changing toothbrushes and the habit of drinking water during meals were associated only with tooth wear, and the preference for hard-bristle toothbrushes and taking vitamin C/aspirin were linked to dentin exposure.

## Supporting Information

S1 File Relevant data underlying the findings described in manuscript. (XLSX)

## References

[1] Eccles JD. Tooth surface loss from abrasion, attrition and erosion. Dent Update. 1982, 9(7):373–374, 376–378, 380–381. 6958629

[2] Ramalho A, Miranda J. The relationship between wear and dissipated energy in sliding systems. Wear. 2006;260:361–367.

[3] Bartlett D, Dugmore C. Pathological or physiological erosion—is there a relationship to age?. Clin Oral Investig. 2008; 12 Suppl 1:S27–S31. 10.1007/s00784-007-0177-1 18228061 PMC2238780

[4] Barbour ME, Rees GD. The role of erosion, abrasion and attrition in tooth wear. J Clin Dent. 2006;17(4): 88–93. 17131710

[5] Muts EJ, van Pelt H, Edelhoff D, Krejci I, Cune M. Tooth wear: a systematic review of treatment options. J Prosthet Dent. 2014;112(4):752–759. 10.1016/j.prosdent.2014.01.018 24721500

[6] Schierz O, Dommel S, Hirsch C, Reissmann DR. Occlusal tooth wear in the general population of Germany: effects of age, sex, and location of teeth. J Prosthet Dent. 2014; 112: 465–471. 10.1016/j.prosdent.2013.12.005 24636759

[7] Kitasako Y, Sasaki Y, Takagaki T, Sadr A, Tagami J. Age-specific prevalence of erosive tooth wear by acidic diet and gastroesophageal reflux in Japan. J Dent. 2015; 43(4): 418–423. 10.1016/j.jdent.2015.02.004 25684603

[8] Vered Y, Lussi A, Zini A, Gleitman J, Sgan-Cohen HD. Dental erosive wear assessment among adolescents and adults utilizing the basic erosive wear examination (BEWE) scoring system. Clin Oral Investig. 2014; 18: 1985–1990. 10.1007/s00784-013-1175-0 24420504

[9] Gupta VV, Asawa K, Bhat N, Tak M, Bapat S, Chaturvedi P et al Assessment of oral hygiene habits, oral hygiene practices and tooth wear among fertilizer factory workers of Northern India: A Cross sectional study. J Clin Exp Dent. 2015; 7(5): e649–e655. 10.4317/jced.52332 26644843 PMC4663069

[10] Zhang J, Du Y, Wei Z, Tai B, Jiang H, Du M. The prevalence and risk indicators of tooth wear in 12- and 15-year-old adolescents in Central China. BMC Oral Heath. 2015; 15(1):120. 10.1186/s12903-015-0104-9 PMC4599587 26453049

[11] Liu B, Zhang M, Chen Y, Yao Y. Tooth wear in aging people: an investigation ofthe prevalence and the influential factors of incisal/occlusal tooth wear in northwest China. BMC Oral Health. 2014;14:65 10.1186/1472-6831-14-65 24902953 PMC4080580

[12] Bartlett D, Ganss C, Lussi A. Basic Erosive Wear Examination (BEWE): a new scoring system for scientific and clinical needs. Clin Oral Investig. 2008 3;12 Suppl 1:S65–68. 10.1007/s00784-007-0181-5 18228057 PMC2238785

[13] Dixon B, Sharif MO, Ahmed F, Smith AB, Seymour D, Brunton PA. Evaluation of the Basic Erosive Wear Examination (BEWE) for use in general dental practice. Br Dent J. 2012;213:E4 10.1038/sj.bdj.2012.670 22878338

[14] Smith BG, Knight JK. An index for measuring the wear of teeth. Br Dent J. 1984;156:435–438. 6590081 10.1038/sj.bdj.4805394

[15] Ganss C, Klimek J, Lussi A. Accuracy and consistency of the visual diagnosis of exposed dentine on worn occlusal/incisal surfaces. Caries Res. 2006; 40: 208–212. 16707868 10.1159/000092227

[16] Mulic A, Tveit AB, Wang NJ, Hove LH, Espelid I, Skaare AB. Reliability of two clinical scoring systems for dental erosive wear. Caries Res. 2010;44:294–299. 10.1159/000314811 20516691

[17] Bartlett D, Lussi A, West NX, Bouchard P, Sanz M, Bourgeois D. Prevalence of tooth wear on buccal and lingual surfaces and possible risk factors in young European adults. J Dent. 2013;41(11):1007–1013. 10.1016/j.jdent.2013.08.018 24004965

[18] Burke FM, Whelton H, Harding M, Crowley E, O'Mullane D, Cronin M et al Fluoridation and tooth wear in Irish adults. Community Dent Oral Epidemiol. 2010; 38: 415–421. 10.1111/j.1600-0528.2010.00550.x 20545717

[19] Muller-Bolla M, Courson F, Smail-Faugeron V, Bernardin T, Lupi-Pegurier L. Dental erosion in French adolescents. BMC Oral Health. 2015; 15(1):147. 26585794 10.1186/s12903-015-0133-4 PMC4653893

[20] González-Aragón Pineda ÁE, Borges-Yáñez SA, Lussi A, Irigoyen-Camacho ME, Angeles Medina F. Prevalence of erosive tooth wear and associated factors in a group of Mexican adolescents. J Am Dent Assoc. 2016; 147(2):92–97. 10.1016/j.adaj.2015.07.016 26562733

[21] Milosevic A, Bardsley PF, Taylor S. Epidemiological studies of tooth wear and dental erosion in 14-year old children in North West England. Part 2: The association of diet and habits. Br Dent J. 2004;197(8):479–483. 15547608 10.1038/sj.bdj.4811747

[22] Ehlen LA, Marshall TA, Qian F, Wefel JS, Warren JJ. Acidic beverages increase the risk of in vitro tooth erosion. Nutr Res. 2008;28(5):299–303. 10.1016/j.nutres.2008.03.001 19083423 PMC2516950

[23] Al-Dlaigan YH, Shaw L, Smith A. Dental erosion in a group of British 14-year-old, school children. Part I: Prevalence and influence of differing socioeconomic backgrounds. Br Dent J. 2001;190(3):145–149. 11236918 10.1038/sj.bdj.4800908

[24] Iwashita H, Tsukiyama Y, Kori H, Kuwatsuru R, Yamasaki Y, Koyano K. Comparative cross-sectional study of masticatory performance and mastication predominance for patients with missing posterior teeth. J Prosthodont Res. 2014; 58: 223–229. 10.1016/j.jpor.2014.04.002 24951162

[25] Gomes SG, Custodio W, Faot F, Cury AA, Garcia RC. Chewing side, bite force symmetry, and occlusal contact area of subjects with different facial vertical patterns. Braz Oral Res. 2011; 25(5): 446–452. 22031059 10.1590/s1806-83242011005000014

[26] Li H, Zou Y, Ding G. Dietary factors associated with dental erosion: a meta-analysis. PLOS ONE. 2012;7(8):e42626 10.1371/journal.pone.0042626 22952601 PMC3432030

[27] McCracken M, O’Neal SJ. Dental erosion and aspirin headache powders: a clinical report. J Prosthodont. 2000; 9(2): 95–98. 11070137 10.1111/j.1532-849x.2000.00095.x

[28] Grace EG, Sarlani E, Kaplan S. Tooth erosion caused by chewing aspirin. J Am Dent Assoc. 2004; 135(7): 911–914. 15354902 10.14219/jada.archive.2004.0337

[29] Bahal P, Djemal S. Dental erosion from an excess of vitamin C. Case Rep Dent 2014 2014: 485387 10.1155/2014/485387 25165584 PMC4137695

[30] Hays GL, Bullock Q, Lazzari EP, Puente ES. Salivary pH while dissolving vitamin C-containing tablets. Am J Dent. 1992; 5(5): 269–271. 1299255

[31] Kakudo Y, Hieda T, Matsuzawa S, Ishida A, Yoshihara M. Relations between brushing force and the number of strokes during tooth brushing in pre-school children and primary school pupils. J Osaka Dent Univ. 1969; 3: 187–199. 5287400

[32] Manly RS, Wiren J, Manly PJ, Keene RC. A method for measurement of abrasion of dentin by toothbrush and dentifrice. J Dent Res. 1965; 44: 533–540. 14304289 10.1177/00220345650440031601

[33] Tate DF, Turner-McGrievy G, Lyons E, Stevens J, Erickson K, Polzien K et al Replacing caloric beverages with water or diet beverages for weight loss in adults: main results of the Choose Healthy Options Consciously Everyday (CHOICE) randomized clinical trial. Am J Clin Nutr. 2012;95(3):555–563. 10.3945/ajcn.111.026278 22301929 PMC3632875

